# Nocturnal sleep duration, clinical activity score, and thyroid functional remission after radioactive iodine therapy in Graves’ disease with thyroid-associated ophthalmopathy

**DOI:** 10.3389/fendo.2026.1954035

**Published:** 2026-10-16

**Authors:** Kehua Liao, Xiaojuan Wei, Yan Chen, Dongyun Meng, Shaozhou Mo, Zeyong Sun, Fengyang Song, Lu Lu, Wentan Huang

**Affiliations:** Department of Nuclear Medicine, The People’s Hospital of Guangxi Zhuang Autonomous Region, Nanning, Guangxi, China

**Keywords:** clinical activity score, Graves’ disease, radioactive iodine therapy, restricted cubic spline, sleep duration, thyroid functional remission, thyroid-associated ophthalmopathy

## Abstract

**Objective:**

To examine whether pretreatment nocturnal sleep duration and Clinical Activity Score (CAS) are associated with thyroid functional remission after radioactive iodine (¹³¹I) therapy in Graves’ disease (GD) with concomitant thyroid-associated ophthalmopathy (TAO). The primary endpoint was thyroid functional remission, not ocular cure.

**Methods:**

We retrospectively reviewed 153 patients at a single center (53 in remission; 100 not in remission), followed for at least 12 months after ¹³¹I therapy. Sleep duration and CAS were pretreatment baseline measures; 20 patients received prednisone before ¹³¹I therapy (discontinued ≥ 3 days prior) and 133 received none; no thyroid-directed or immunosuppressive treatment was given during follow-up. Remission was coded using the response categories of the 2021 Chinese Society of Nuclear Medicine ¹³¹I treatment guideline (euthyroidism or hypothyroidism vs. partial or ineffective response). Missing continuous values were multiply imputed (20 chains). Analyses included false discovery rate (FDR)–controlled univariate screening, cross-validated least absolute shrinkage and selection operator (LASSO) selection, post-selection logistic regression, Firth sensitivity analysis, interaction tests, and mutually adjusted restricted cubic splines (RCS).

**Results:**

No patient sleeping less than 6h achieved remission [0/31; 95% confidence interval (CI) 0%–9.2%]. After FDR adjustment, GD duration, TAO duration, sleep duration, and CAS remained associated with remission. LASSO (one-standard-error rule, *C* = 0.142) retained GD duration, sleep duration, and CAS. In the post-selection model, the odds ratio (OR) per month of GD duration was 0.991 (95% CI 0.979–1.003; *P* = 0.152); per additional sleep hour, 6.13 (95% CI 3.16–11.91; *P* < 0.001; Firth OR 5.64, 95% CI 2.99–10.63); and per CAS point, 0.64 (95% CI 0.48–0.86; *P* = 0.003). The CAS × sleep interaction was not significant (Wald *P* = 0.096; likelihood-ratio *P* = 0.086). Mutually adjusted RCS supported non-linearity for CAS (*P* < 0.001) but not for sleep (*P* = 0.585).

**Conclusions:**

Longer pretreatment nocturnal sleep and lower CAS were associated with thyroid functional remission after ¹³¹I therapy in this single-center cohort, with consistent directions across sensitivity models. These retrospective, post-selection associations require prospective validation; the absence of remission below 6h and the fitted threshold effects are exploratory *post hoc* observations, not validated thresholds.

## 1 Introduction

Thyroid-associated ophthalmopathy (TAO) is the most common extrathyroidal manifestation of Graves’ disease (GD), affecting 25%–50% of patients (1, 2). Orbital fibroblasts co-express thyrotropin (TSH) receptor and insulin-like growth factor-1 receptor, which drives orbital inflammation, hyaluronic acid deposition, and adipogenesis (2, 3). Clinical features range from eyelid retraction and proptosis to diplopia and dysthyroid optic neuropathy. About 3%–5% of patients progress to sight-threatening disease (1, 3).

Management of TAO may include antithyroid drugs (ATDs) or ¹³¹I for hyperthyroidism, glucocorticoid or other immunomodulatory therapy, orbital radiotherapy, and surgery, according to disease activity and severity (4). Teprotumumab is approved for active disease in some settings (5) but was not available at our center during the study period. Treatment courses and ocular outcomes vary widely (6, 7). Observational associations can identify candidate predictors, but they cannot establish whether changing a given factor would improve response.

Sleep insufficiency and other clinical factors have been linked to poor ¹³¹I outcomes in GD in a previous single-center analysis (8). Sleep–immune pathways are biologically plausible, yet they do not prove causality. The Clinical Activity Score (CAS) is a standard measure of inflammatory activity (9). Age, sex, smoking, thyroid function, thyrotropin receptor antibody (TRAB), and CAS have also been related to TAO prognosis (7, 10, 11). Whether these relationships are non-linear remains unclear.

Restricted cubic splines (RCSs) can describe non-linear relationships without imposing an arbitrary cut-off (12, 13). In small observational samples, the fitted curve is easily distorted by sparse data and confounding. Spline peaks should therefore not be treated as clinical thresholds.

We therefore examined whether pretreatment nocturnal sleep duration and CAS were associated with thyroid functional remission after ¹³¹I therapy, and whether their dose–response relationships departed from linearity.

## 2 Methods

### 2.1 Study population

We retrospectively reviewed 153 patients with GD and concomitant TAO of varying severity, all with a history of ATD treatment. All received initial ¹³¹I therapy at the People’s Hospital of Guangxi Zhuang Autonomous Region between January 2020 and June 2025.

Inclusion criteria: (1) confirmed diagnosis of Graves’ hyperthyroidism and consent to first-time ¹³¹I therapy as routine clinical care; (2) concomitant TAO of any severity; (3) ATDs withdrawn before ¹³¹I therapy for at least 1 week (methimazole) or 4 weeks (propylthiouracil), with hyperthyroidism confirmed at the time of treatment; (4) ¹³¹I prescribed under the institutional dosing protocol of 70–120 μCi per gram of thyroid tissue; and (5) ability to communicate autonomously, without psychological or psychiatric disorders.

Exclusion criteria: (1) pre-existing ocular comorbidities including high myopia, glaucoma, or significant pterygium; (2) history of ocular surgery or orbital radiotherapy; (3) hyperthyroid heart disease or other underlying cardiac conditions with cardiac insufficiency; and (4) severe hepatic or renal impairment.

Pretreatment protocol: At our center, patients with severe infiltrative TAO in the active progressive phase who require immunosuppressive therapy may receive glucocorticoids or other immunomodulatory agents before ¹³¹I therapy and proceed to radioiodine only after the disease has stabilized. In the present consecutive cohort, chart review confirmed that 20 patients with more severe ocular symptoms received varying doses of prednisone for 8–16 weeks before ¹³¹I therapy, discontinued at least 3 days before treatment; the remaining 133 patients received no glucocorticoids or other immunosuppressive therapy before ¹³¹I therapy.

Patients were monitored for at least 12 months after ¹³¹I therapy, with outcome status determined from the thyroid function assessment at 12 months or later. No patient received ATDs, ¹³¹I retreatment, thyroid surgery, glucocorticoids, or other immunosuppressive therapy during follow-up. Treatment response was classified using four thyroid-function response categories conceptually consistent with the 2016 American Thyroid Association (ATA) guidelines (14) and specified in the 2021 Chinese Society of Nuclear Medicine guideline for ¹³¹I treatment of Graves’ hyperthyroidism (15); the criteria require at least 6 months of follow-up, and all patients in this cohort were followed for at least 12 months. Euthyroidism (complete remission) was defined as complete resolution of hyperthyroid symptoms and signs with serum free thyroxine (FT4) within the reference range. Hypothyroidism was defined as hypothyroid symptoms or signs with serum FT4 below the reference range and TSH above it; all patients classified as hypothyroid initiated levothyroxine replacement therapy. Partial remission was defined as reduced hyperthyroid symptoms with partial resolution of signs and decreased but not normalized serum FT4. An ineffective response was defined as unimproved or worsening symptoms and signs with serum FT4 essentially unchanged. Euthyroidism or hypothyroidism was classified as remission (*n* = 53). Partial remission or an ineffective response was classified as non-remission (*n* = 100). Outcome classification was performed by physicians who had not participated in the extraction of the exposure variables. Euthyroidism and hypothyroidism were combined as functional remission because both indicate resolution of hyperthyroidism after ¹³¹I: hypothyroidism is an expected outcome of successful ¹³¹I therapy, is readily managed with levothyroxine replacement, and is grouped with euthyroidism as a successful response in the guideline framework applied here (14, 15).

This study was approved by the Ethics Committee of the People’s Hospital of Guangxi Zhuang Autonomous Region (Approval No. KY-GZR-2025-035). Fifteen participants were younger than 18 years. ¹³¹I is an established treatment for adolescents with GD at our center, and these patients formed part of the consecutive treatment series; a sensitivity analysis excluding them yielded consistent estimates (Section 3.2). The Ethics Committee waived written informed consent, including guardian consent for minors, because of the retrospective design (see Declarations).

### 2.2 Clinical variables

We collected age, sex, smoking history, self-reported nocturnal sleep duration, GD duration, TAO duration, free triiodothyronine (FT3), FT4, thyroid peroxidase antibody (TPOAB), TRAB, CAS assessed on the European Group on Graves’ Orbitopathy (EUGOGO) 7-point scale (4), proptosis, palpebral fissure height, and thyroid nodule status. Age, sex, smoking history, sleep duration, GD duration, TAO duration, FT3, FT4, TPOAB, TRAB, and thyroid nodule status were recorded within 1 week before ¹³¹I therapy; the ocular measures (CAS, proptosis, and palpebral fissure height) were assessed at the pretreatment ophthalmic evaluation before any glucocorticoid intervention. Nocturnal sleep duration and CAS were assessed as pretreatment baseline variables and were traceable in the medical records. Thyroid nodule presence was analyzed as a binary variable (absent versus present). Administered ¹³¹I activity was protocolized at 70–120 μCi per gram of thyroid tissue in all patients; because dosing followed this uniform institutional protocol, per-patient activities were not extracted or analyzed as covariates. Antithyroid-drug washout was standardized by protocol (Section 2.1) rather than modeled. Baseline TSH was not analyzed because values in overt thyrotoxicosis were frequently below the assay’s lower reporting limit; thyroid volume and radioiodine uptake were not uniformly recorded in extractable form. Glucocorticoid exposure before ¹³¹I therapy was ascertained by chart review in all 153 patients (Section 2.1).

### 2.3 Missing data handling

A few values were missing (FT3: 1; FT4: 1; TPOAB: 3; TRAB: 6; proptosis: 1; palpebral fissure height: 1; smoking history: 1). Continuous variables were imputed with 20 multiple imputations using Bayesian Ridge regression (seeds 2026–2045); the single missing smoking value was filled with the mode. Values reported above an assay upper limit (one FT3 and one FT4 value) were kept at that limit and flagged. We confirmed that no originally observed value was altered by imputation. Estimates involving imputed variables (Section 3.2) were pooled across imputations with Rubin’s rules. Sleep duration, CAS, GD duration, TAO duration, thyroid nodule presence, and the outcome were completely observed; the 20 completed data sets are therefore identical for every variable entering the multivariable, stratified, interaction, and spline models. Those analyses were run on a single completed data set and yield estimates numerically identical to pooled ones.

### 2.4 Statistical analysis

The analysis proceeded in three sequential steps. All tests were two-sided with α = 0.05. This was a consecutive retrospective series; no *a priori* sample-size calculation was performed.

Step 1: Screening and selection. Univariate logistic regression was performed for 14 clinical variables, and Benjamini–Hochberg false discovery rate-adjusted q values were reported. Variables with *q* < 0.05 underwent variance inflation factor (VIF) assessment and entered L1-penalized least absolute shrinkage and selection operator (LASSO) regression (16); this pre-screening step reused the same data and is heuristic. Standardization was performed within each training fold; stratified fivefold cross-validation used shuffled folds with a fixed seed; and the one-standard-error (1-SE) rule selected the penalty. Across 50 repeated cross-validation runs with different fold partitions, sleep and CAS were retained in every run and GD duration in 64% of runs (1-SE C range 0.067–0.359). Variables with non-zero coefficients then entered an unpenalized logistic model. We report odds ratios (ORs), Wald 95% confidence intervals (CIs), and *P* values from this model as post-selection estimates. Because selection and estimation used the same sample, we do not claim internally validated prediction performance. Given complete separation at short sleep durations, we also fitted Firth bias-reduced logistic regression for the same covariate set as a sensitivity analysis. A second sensitivity model adjusted the post-selection covariates for the prespecified clinical confounders age, sex, and smoking.

Step 2: Stratified and interaction analysis. Patients were stratified by nocturnal sleep duration (<6h and 6h–8h); the 6h cut point was chosen after inspecting the outcome distribution and was not prespecified. Within each stratum, logistic regression of remission on CAS was performed. The continuous CAS × sleep interaction coefficient was evaluated using a Wald test; nested models with and without the interaction were additionally compared using a likelihood-ratio test (LRT). Both results are reported.

Step 3: Mutually adjusted RCS analysis. Natural cubic splines with one internal knot at the median (P50) and boundary knots at P10 and P90 (2 degrees of freedom per variable) were fitted simultaneously for sleep and CAS (12, 13). Sleep curves were adjusted for the CAS spline, and CAS curves for the sleep spline; GD duration was not included in the spline model. ORs were plotted relative to each variable’s median while holding the other at its median, and curves were displayed within the boundary knots to avoid extrapolation. Non-linearity was tested by likelihood-ratio comparison with a model that replaced the tested spline with a linear term. As a robustness check, the mutually adjusted splines were refitted with two (P25/P75) and three (P25/P50/P75) interior knots.

Analyses were performed in Python 3.9 (statsmodels 0.14; scikit-learn 1.6) and R 4.6 (natural cubic splines). Firth logistic regression was implemented in Python with iterative bias-reduced score equations, and Wald intervals were computed at the Firth estimates. Fixed random seeds were used. The binary outcome was remission versus non-remission.

## 3 Results

### 3.1 Baseline characteristics

Among 153 patients, 53 (34.6%) achieved thyroid functional remission and 100 (65.4%) did not. Compared with patients without remission, those in remission had longer pretreatment nocturnal sleep, lower CAS, and shorter median GD and TAO durations, whereas thyroid nodule prevalence did not differ between groups (**Table 1**). Ocular symptoms were recorded as progressed after treatment in 37 patients (24.2%).

**Table 1:** Baseline characteristics of remission versus non-remission groups.

Variable	Non-remission (*n* = 100)	Remission (*n* = 53)	*P*
Age (years)	37.6 ± 13.0	36.0 ± 14.1	0.473
Female, *n* (%)	79 (79.0%)	44 (83.0%)	0.670
Smoking history, *n* (%)	33 (33.0%)	11 (20.8%)	0.135
Thyroid nodule, *n* (%)	35 (35.0%)	22 (41.5%)	0.484
GD duration (months)	24.0 [6.0–61.5]	6.0 [2.0–24.0]	< 0.001
TAO duration (months)	10.0 [5.0–24.5]	5.0 [3.0–8.0]	< 0.001
Sleep duration (h)	6.0 [5.0–7.0]	8.0 [7.0–8.0]	< 0.001
CAS score	4.0 [3.0–4.0]	2.0 [0.0–2.0]	< 0.001
Proptosis (mm)	15.1 ± 2.4	15.6 ± 2.7	0.174
Palpebral fissure (mm)	10.5 ± 1.7	11.1 ± 1.9	0.097
FT3 (pmol/L)	28.2 ± 12.6	25.1 ± 11.2	0.139
FT4 (pmol/L)	57.9 ± 18.1	56.8 ± 17.4	0.770
TPOAB (IU/ml)	396.9 ± 402.5	409.5 ± 384.7	0.614
TRAB (IU/L)	23.9 ± 12.6	20.5 ± 10.7	0.059

Data are *M* ± SD, median [IQR], or *n* (%). GD duration, TAO duration, sleep duration, and CAS are summarized as median [IQR]. *P* values are from Fisher’s exact tests for binary variables, Mann–Whitney U tests for GD duration, TAO duration, sleep duration, and CAS, and Welch tests for other continuous variables. *M* ± SD entries are averaged across the 20 imputed data sets; *P* values were computed on the first imputed data set.

### 3.2 Univariate logistic regression and least absolute shrinkage and selection operator selection

Univariate logistic regression results are shown in **Table 2**. Four variables retained *q* < 0.05 after Benjamini–Hochberg adjustment: GD duration (*q* = 0.029), TAO duration (*q* = 0.024), sleep duration (*q* < 0.001), and CAS (*q* < 0.001). VIF values ranged from 1.13 to 1.44.

**Table 2:** Univariate logistic regression; raw *P* values and Benjamini–Hochberg FDR-adjusted *q* values are shown.

Variable	OR	95% CI	*P*; FDR q
Age (per year)	0.991	0.966–1.016	0.459; 0.585
Female sex	1.300	0.548–3.082	0.552; 0.644
Smoking history	0.532	0.243–1.164	0.114; 0.229
Thyroid nodule (present)	1.318	0.665–2.611	0.429; 0.585
GD duration (per month)	0.986	0.975–0.996	0.008; 0.029
TAO duration (per month)	0.957	0.927–0.987	0.005; 0.024
Sleep duration (per hour)	7.648	4.005–14.605	< 0.001; < 0.001
CAS score (per point)	0.529	0.408–0.685	< 0.001; < 0.001
Proptosis (per mm)	1.096	0.959–1.252	0.179; 0.278
Palpebral fissure (per mm)	1.188	0.977–1.444	0.084; 0.229
FT3 (per pmol/L)	0.979	0.952–1.007	0.133; 0.233
FT4 (per pmol/L)	0.997	0.978–1.015	0.719; 0.774
TPOAB (per IU/ml)	1.000	0.999–1.001	0.852; 0.852
TRAB (per IU/L)	0.976	0.949–1.005	0.101; 0.229

LASSO with shuffled stratified fivefold cross-validation selected *C* = 0.142 by the 1-SE rule. GD duration (standardized coefficient −0.061), sleep duration (+1.455), and CAS (−0.419) retained non-zero coefficients; TAO duration was shrunk to zero (**Figure 1**).

**Figure 1:**
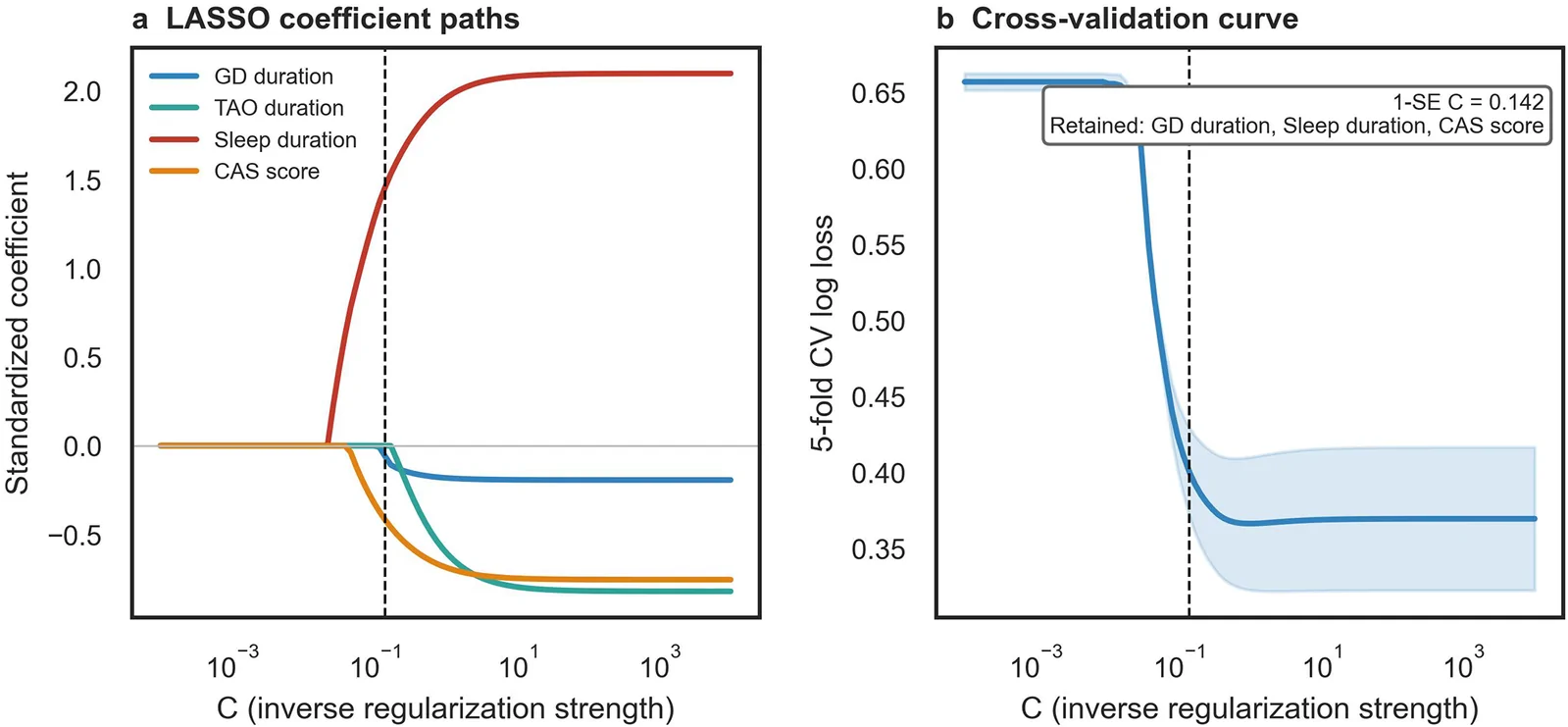
LASSO variable selection. **(a)** Coefficient paths across the regularization grid. **(b)** Cross-validation curve. Standardization was performed within shuffled stratified cross-validation folds. At the 1-SE value C = 0.142, GD duration, sleep duration, and CAS retained non-zero coefficients; TAO duration was zero. n = 153.

In the multivariable model after selection (**Table 3**; **Figure 2**), the OR per month of GD duration was 0.991 (95% CI 0.979–1.003; *P* = 0.152), equivalent to 0.90 (95% CI 0.77–1.04) per year. The OR per additional hour of nocturnal sleep was 6.13 (95% CI 3.16–11.91; *P* < 0.001). The OR per CAS point was 0.64 (95% CI 0.48–0.86; *P* = 0.003). These estimates derive from selection and fitting in the same sample and should be read as post-selection coefficients. In Firth bias–reduced logistic regression with the same covariates, the sleep OR was 5.64 (95% CI 2.99–10.63; *P* < 0.001), the CAS OR was 0.65 (95% CI 0.49–0.87; *P* = 0.003), and the GD-duration OR was 0.993 (95% CI 0.982–1.004; *P* = 0.203), supporting directional robustness despite separation at short sleep. In a model additionally adjusted for age, sex, and smoking, the estimates were materially unchanged: sleep OR 7.57 (95% CI 3.58–16.01; *P* < 0.001), CAS OR 0.61 (95% CI 0.45–0.83; *P* = 0.001), and GD-duration OR 0.991 (95% CI 0.980–1.002; *P* = 0.113). The age-, sex-, and smoking-adjusted model was specified *a priori* as the clinically prespecified confounder model. Adding thyroid nodule presence to the post-selection model likewise left the estimates essentially unchanged (sleep OR 6.79, 95% CI 3.33–13.81; CAS OR 0.63, 95% CI 0.47–0.84; GD-duration OR 0.988, 95% CI 0.975–1.002); nodule presence was nominally associated with remission only after adjustment (OR 2.99, 95% CI 1.01–8.78; *P* = 0.047; Firth OR 2.75, *P* = 0.055). Excluding the 15 participants younger than 18 years (*n* = 138; 47 in remission) yielded consistent estimates: sleep OR 5.53 (95% CI 2.76–11.08; *P* < 0.001), CAS OR 0.61 (95% CI 0.44–0.84; *P* = 0.002), and GD-duration OR 0.990 (95% CI 0.978–1.003; *P* = 0.119).

**Table 3:** Multivariable logistic regression after selection.

Variable	LASSO coefficient	OR	95% CI (Wald)	*P*
GD duration (per month)	-0.061	0.991	0.979–1.003	0.152
Sleep duration (per hour)	1.455	6.13	3.16–11.91	< 0.001
CAS score (per point)	-0.419	0.64	0.48–0.86	0.003

LASSO coefficients are standardized coefficients from the 1-SE model (*C* = 0.142). ORs, Wald CIs, and *P* values are from the subsequent unpenalized model and are post-selection estimates conditional on selection in the same cohort. Firth sensitivity ORs for the same covariates were 5.64 per sleep hour and 0.65 per CAS point. Estimates are post-selection coefficients from a model fitted in the same sample used for selection and should be interpreted accordingly (17); the prespecified confounder model additionally adjusting for age, sex, and smoking is reported in Section 3.2.

**Figure 2:**
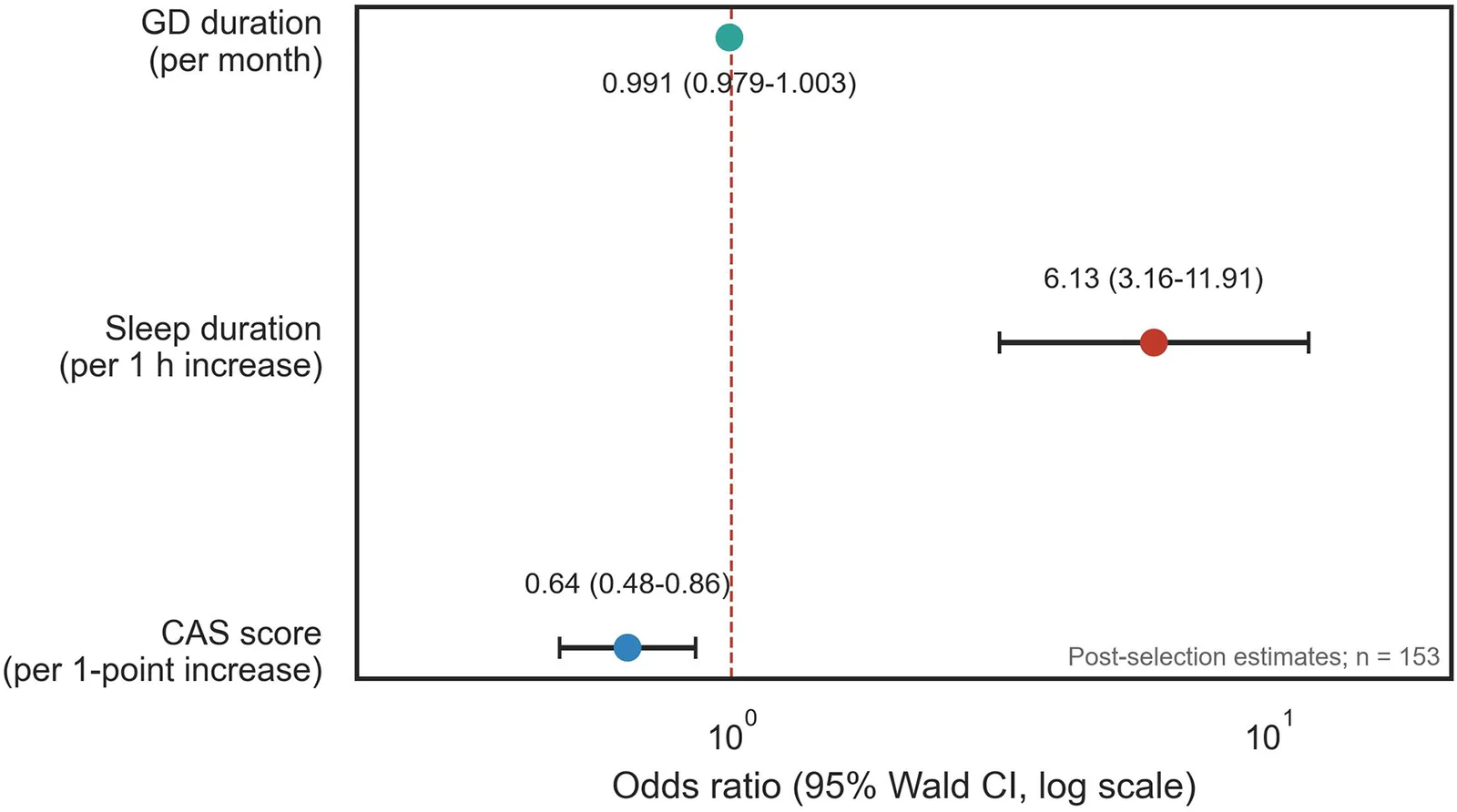
Multivariable logistic regression after selection. Points show ORs and bars show Wald 95% CIs. Estimates are conditional on selection in the same sample. Dashed line: OR = 1. *n* = 153.

### 3.3 Clinical activity score–outcome stratified analysis by sleep duration

Results stratified by nocturnal sleep duration are presented in **Table 4**. No patient reported more than 8h of sleep, so two strata were analyzed.

**Table 4:** CAS–remission logistic regression stratified by nocturnal sleep duration.

Sleep stratum	*n*	Remission rate	CAS OR	95% CI	P
< 6h	31	0%†	NE	NE	NE
6–8h	122	43.4%	0.57	0.44–0.74	< 0.001

†Complete separation (0% remission rate); NE = not estimable. Continuous CAS × sleep interaction: Wald *P* = 0.096; nested-model LRT *P* = 0.086.

<6h (*n* = 31): No patient achieved remission (0/31; 95% CI 0%–9.2%), so the CAS effect could not be estimated reliably.

6h–8h stratum (*n* = 122): Each 1-point increase in CAS was associated with an OR of 0.57 (95% CI 0.44–0.74; *P* < 0.001).

We found no significant CAS × sleep interaction. The Wald test for the interaction coefficient yielded *P* = 0.096, and the nested-model LRT yielded *P* = 0.086 (**Figure 3**).

**Figure 3:**
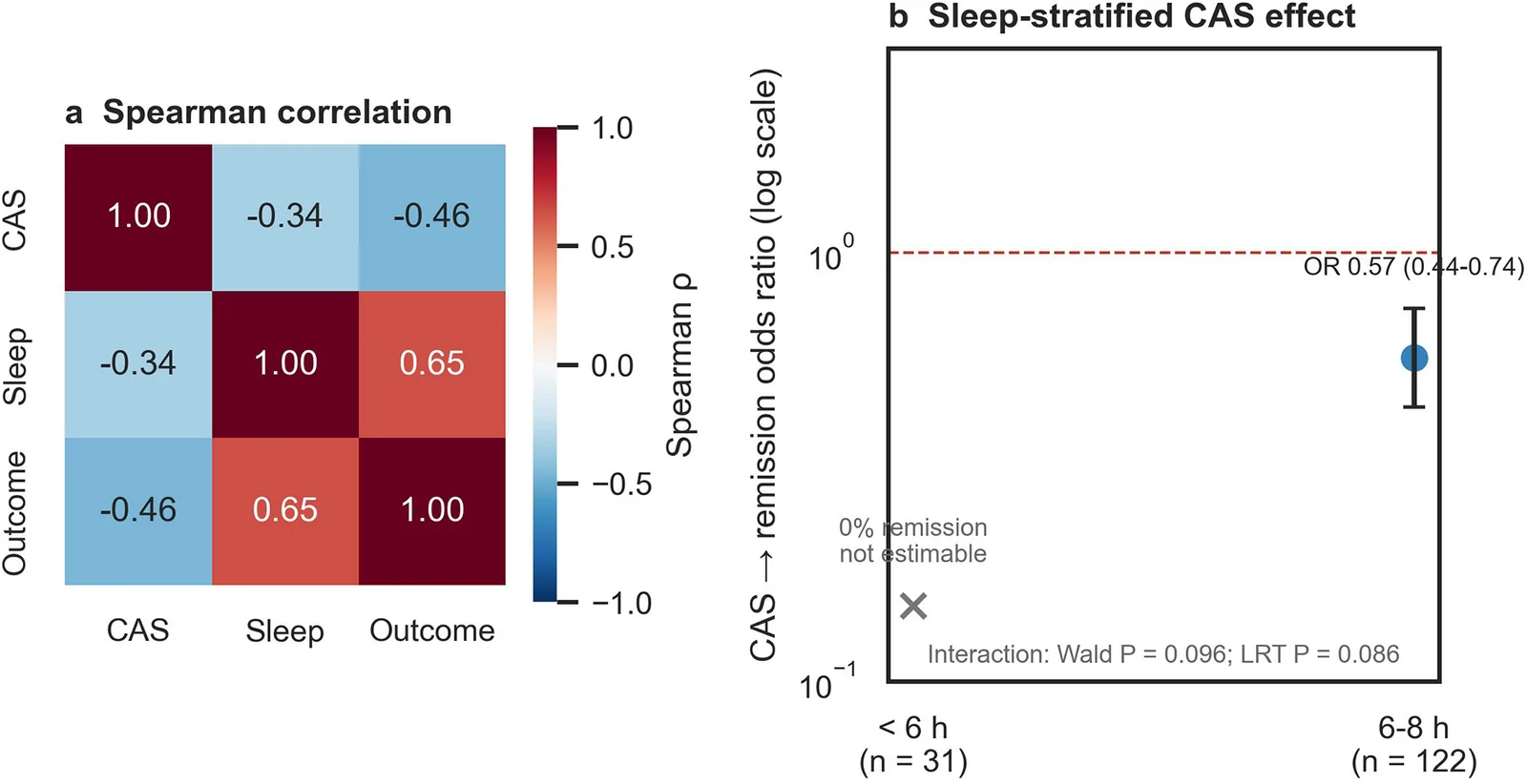
Correlation and stratified analysis. **(a)** Spearman heatmap for Clinical Activity Score (CAS), sleep, and remission outcome. **(b)** CAS-to-remission OR by sleep stratum. The <6h estimate was not estimable because no patient achieved remission. Interaction: Wald *P* = 0.096; LRT *P* = 0.086. *n* = 153.

### 3.4 Restricted cubic spline analysis

The mutually adjusted RCS curves are shown in **Figure 4**.

**Figure 4:**
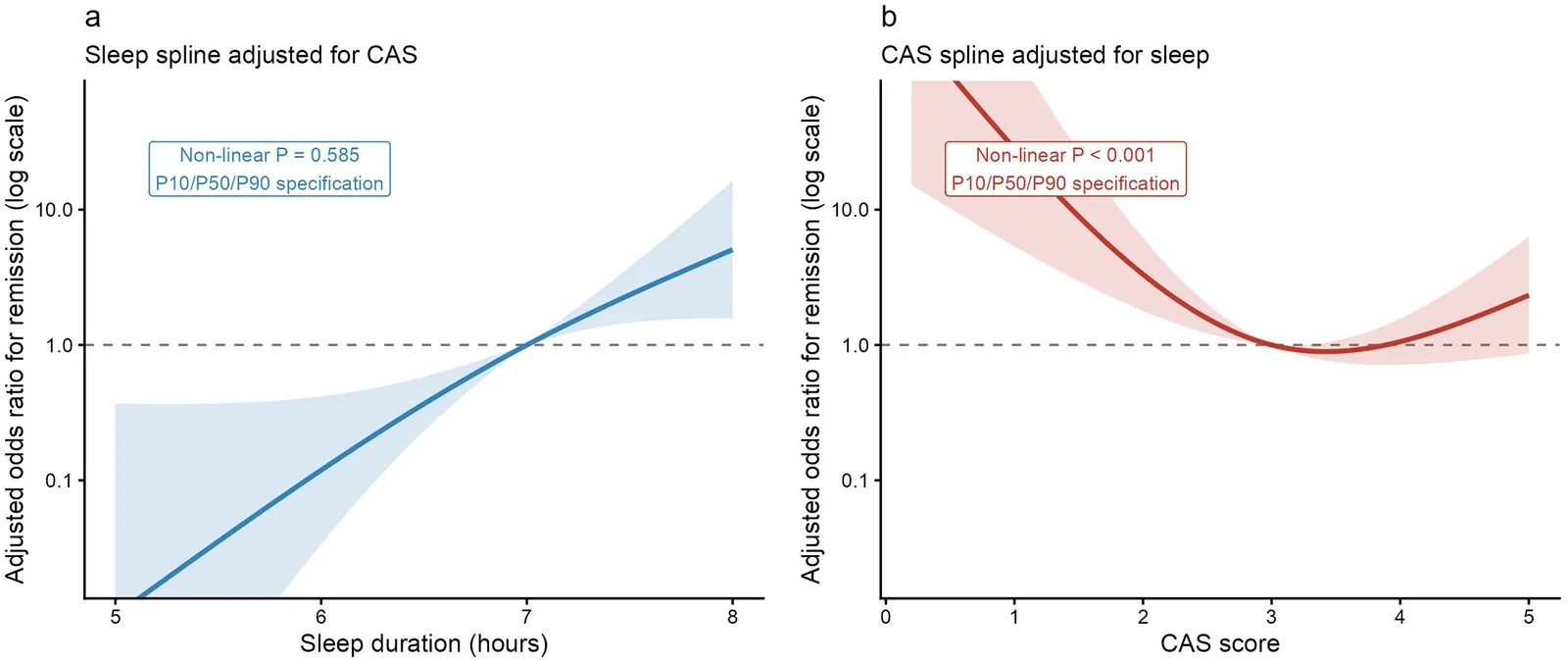
Mutually adjusted restricted cubic spline curves for **(a)** nocturnal sleep duration and **(b)** the Clinical Activity Score (CAS) (one internal knot at P50; boundary knots at P10 and P90; curves displayed within the boundary knots). Sleep is adjusted for the CAS spline and CAS for the sleep spline. Solid lines show ORs relative to the median and shaded regions show 95% Wald CIs. Non-linear P = 0.585 for sleep and P < 0.001 for CAS. n = 153.

For nocturnal sleep duration, after adjustment for CAS, the non-linearity test yielded *P* = 0.585. With only 31 patients and no remission events below 6h, this test has limited power; we interpret it as failing to detect non-linearity rather than excluding it. The test remained non-significant with two (*P* = 0.727) or three (*P* = 0.574) interior knots. Across the observed range, the association was approximately linear; using the median of 7h as reference, each 1h reduction in sleep was associated with a graded increase in the odds of non-remission (Section 3.6).

For CAS, after adjustment for sleep, non-linearity remained (*P* < 0.001) and persisted with two or three interior knots (both *P* < 0.001). The curve was non-monotonic, and few patients had high CAS. Because CAS was assessed before any glucocorticoid intervention (Section 2.2), the curve shape is unlikely to be explained by steroid indication confounding alone (Section 4.3). Sparse high-CAS cells and the single-center design still limit mechanistic interpretation.

### 3.5 Observed sleep–outcome distribution

Because sleep values were discrete and no remission occurred below 6h, we emphasize the unadjusted remission rates at each reported sleep duration (**Table 5**; **Figure 5**) as the primary view of the sleep association. Continuous ORs are secondary and sensitive to separation.

**Table 5:** Remission rates by reported nocturnal sleep duration.

Sleep duration (h)	*n*	Non-remission, *n*	Remission, *n*	Remission rate	% of non-remission group	% of remission group
4.0	7	7	0	0.0%	7.0%	0.0%
5.0	24	24	0	0.0%	24.0%	0.0%
6.0	39	36	3	7.7%	36.0%	5.7%
6.5	3	3	0	0.0%	3.0%	0.0%
7.0	43	23	20	46.5%	23.0%	37.7%
8.0	37	7	30	81.1%	7.0%	56.6%

Percentages in the last two columns use all patients without remission (*n* = 100) and all patients with remission (*n* = 53) as denominators. Values are unadjusted.

**Figure 5:**
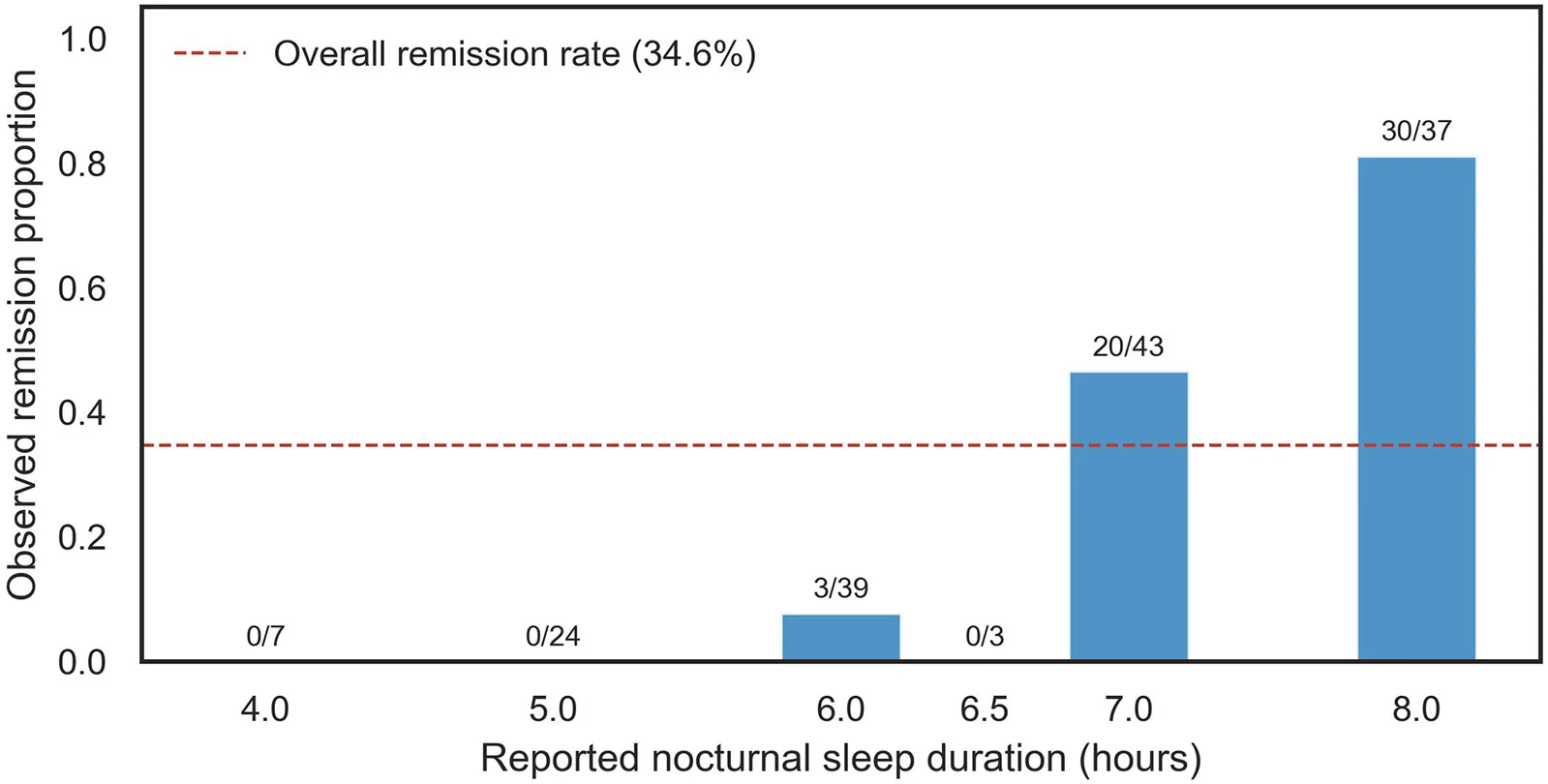
Remission proportion at each reported nocturnal sleep duration. Labels show remission/total patients. The dashed line is the overall remission proportion. Values are unadjusted. n = 153.

No patient achieved remission at 4h, 5h, or 6.5h of sleep. Remission occurred in 3 of 39 patients at 6h, 20 of 43 at 7h, and 30 of 37 at 8h.

**Table 5** and **Figure 5** are unadjusted. Sleep quality, sleep disorders, and objective sleep measures were not available. The complete absence of remission below 6h accounts for much of the large continuous sleep OR and motivates the Firth sensitivity analysis above. Accordingly, the continuous per-hour sleep OR should not be interpreted as a dose-response effect estimate: sleep was self-reported at only six discrete values, rounding is likely, and complete separation below 6h inflates the estimate despite Firth attenuation.

### 3.6 Threshold effect analysis

Two-piecewise logistic regression was used to screen for inflection points in the sleep–remission and CAS–remission relationships; for each variable, a two-piecewise logistic model was fitted over a grid of candidate cut points and the cut point maximizing the model likelihood was selected; ORs were then estimated by separate logistic regression within each resulting segment. This analysis was performed after inspection of the data, was not prespecified, and is reported as exploratory.

Results are shown in **Table 6**. For nocturnal sleep duration, the maximum-likelihood inflection point was 7.0h: among patients sleeping ≤7h (*n* = 116), each additional hour of sleep was associated with an OR of 12.78 (95% CI 3.57–45.70; *P* < 0.001). Above 7h (*n* = 37), no dose gradient could be estimated because all patients reported exactly 8h of sleep; remission in this group was 81.1% (30 of 37), consistent with a plateau above the inflection point. For CAS, the inflection point was 4 points: at CAS ≤ 4 (*n* = 128), each additional point was associated with an OR of 0.21 (95% CI 0.12–0.36; *P* < 0.001); above 4 points (*n* = 25), the OR reversed to 8.43 (95% CI 1.44–49.44; *P* = 0.018). The high-CAS segment included only 25 patients with a wide confidence interval, and the reversal may reflect confounding by intensified treatment (Section 4.3); the fitted inflection points are exploratory and require external validation.

**Table 6:** Exploratory two-piecewise (threshold effect) analysis of nocturnal sleep duration and CAS.

Variable and segment	*n*	OR (95% CI)	*P* value
Nocturnal sleep duration ≤ 7h	116	12.78 (3.57–45.70)	< 0.001
Nocturnal sleep duration > 7h	37	NE	—
CAS ≤ 4 points	128	0.21 (0.12–0.36)	< 0.001
CAS > 4 points	25	8.43 (1.44–49.44)	0.018

Inflection points (7.0h for sleep; 4 points for CAS) were selected by maximizing the two-piecewise model likelihood over observed values; segment ORs are from separate logistic regressions within each segment. The analysis was *post hoc* and is not externally validated. NE = not estimable (all patients above 7h reported exactly 8h of sleep; remission 30 of 37, 81.1%).

## 4 Discussion

### 4.1 Principal findings

Pretreatment nocturnal sleep duration and CAS were associated with thyroid functional remission. Both associations persisted after multivariable selection, whereas GD duration was no longer significant at α = 0.05. Mutually adjusted RCS favored a non-linear pattern for CAS but not for sleep. We retain the CAS non-linearity as a principal finding in this consecutive cohort, although the fitted shape does not establish a validated threshold.

### 4.2 Sleep duration and ¹³¹I treatment outcomes

A previous single-center analysis linked sleep insufficiency with suboptimal ¹³¹I outcomes (8). Radioiodine therapy may also influence the course of Graves’ orbitopathy (18, 19). Here, sleep was a pretreatment baseline measure, but sleep quality was not captured and administered activity was not modeled, so the association cannot be attributed to a specific mechanism.

Nocturnal sleep duration showed the largest modeled association (OR = 6.13 per hour; Firth OR = 5.64). Sleep–immune pathways are biologically plausible (20–22), but they do not exclude measurement bias, reverse causation, or residual confounding. Hyperthyroidism itself disturbs sleep, so short reported sleep may mark more severe thyrotoxicosis rather than an independent risk factor. This bidirectionality is well documented: thyrotoxicosis is a recognized cause of sleep dysfunction, and in a series of 137 patients with GD summarized in a recent review, 66.4% reported difficulty falling asleep (23). We therefore interpret the sleep association as compatible with both a contributory role of sleep and a marker of disease severity. Similar FT3, FT4, and TRAB levels between groups only partly address this pathway, because thyroid volume, radioiodine uptake, and administered activity were not included in the models. No patient sleeping less than 6h achieved remission, and most patients in remission reported 8h of sleep. **Table 5** therefore provides the most transparent summary of the sleep–remission gradient under complete separation.

After adjustment for CAS, the sleep curve did not support non-linearity (*P* = 0.585). Sleep was reported at only six discrete values between 4h and 8h, and self-report may have rounded the values. These findings do not define a minimum sleep target or show that extending sleep improves response.

### 4.3 Non-linear clinical activity score association and its uncertainty

CAS, proptosis, and palpebral fissure height capture related but distinct aspects of TAO. CAS tracks clinical inflammatory activity, whereas proptosis and fissure height can also reflect structural change. That CAS was selected in this study does not prove that inflammation is the main driver of response.

The mutually adjusted spline suggested that a single linear CAS OR did not fully describe the association. The curve was fitted in the same modest sample, and high CAS values were uncommon. The non-monotonic shape is therefore not evidence for a CAS cut-off or a protective high-CAS effect.

Indication confounding by glucocorticoid therapy is often a concern when CAS is high (4, 24). CAS was assessed before any glucocorticoid intervention, and the 20 patients who received prednisone before ¹³¹I therapy had discontinued it at least 3 days before treatment (Section 2.1); steroid indication confounding is therefore an unlikely primary explanation for the CAS association. Glucocorticoid pretreatment was not modeled as a covariate, however, and residual confounding by factors not entered into the models, including administered ¹³¹I activity, remains possible.

Segmented CAS ORs are reported only within the exploratory threshold analysis (Section 3.6); because no cut point was prespecified or independently validated, they carry no confirmatory weight.

Flexible curves fitted in small samples need external validation before they can guide clinical decisions.

### 4.4 Clinical activity score–sleep interaction

Neither the Wald test (*P* = 0.096) nor the LRT (*P* = 0.086) supported a CAS × sleep interaction, although these tests were likely underpowered to detect a moderate interaction at this sample size. Complete separation below 6h further limits subgroup estimates. These results do not identify a treatment subgroup or support a change in ¹³¹I dose.

### 4.5 Comparison with existing literature

LASSO offered a reproducible way to reduce the number of covariates (16). Sex, age, and smoking remain important clinical modifiers of TAO and should be interpreted alongside sleep and CAS (25). Selection within the same sample does not replace confounder adjustment defined in advance.

The mutually adjusted splines avoided analyzing sleep and CAS in isolation. Recent cohorts further underscore the need for careful radioiodine-related ocular risk stratification (19, 26). Our aim was to examine pretreatment predictors of thyroid functional remission, not to validate a model; post-selection inference remains a concern in small samples (17).

### 4.6 Clinical implications

Pretreatment sleep and CAS remain candidates for prospective study. Clinical decisions should continue to follow EUGOGO guidance (4). Evidence on glucocorticoid cover after radioiodine is summarized elsewhere (24) and applied only to the 20 patients who received pretreatment prednisone.

At present, the priority is to replicate these pretreatment associations in models that include charted administered activity and objective sleep measures. The findings are not suitable for clinical risk stratification.

### 4.7 Limitations

This study has major limitations. First, the primary endpoint was thyroid functional remission, not a composite ocular cure; CAS and other eye signs were predictors rather than outcome components, so the findings should not be read as predictors of TAO resolution. Post-therapy ocular status was not systematically graded; ocular symptoms were recorded as progressed in 37 patients (24.2%), but no standardized post-therapy CAS or severity grading was available, and the study cannot inform the ocular prognosis or ocular safety of ¹³¹I in TAO. Second, although sleep and CAS were documented as pretreatment baseline measures in the medical records, sleep was self-reported at only six discrete values, and no patient sleeping less than 6h achieved remission, which inflates the continuous sleep OR despite Firth attenuation. Third, established determinants of ¹³¹I response (individual administered activity, thyroid volume, and radioiodine uptake) were not available for modeling, and antithyroid-drug washout, although standardized by protocol (Section 2.1), was not modeled; adherence, sleep disorders, alcohol use, and socioeconomic factors were likewise not analyzed. Although administered activity was protocolized at 70–120 μCi/g in all patients, protocolized dosing does not remove confounding, because thyroid volume and uptake determine the absorbed dose and vary across patients. We regard the resulting residual confounding as a core limitation. Fourth, 20 patients with more severe TAO received prednisone before ¹³¹I therapy per institutional protocol, and glucocorticoid pretreatment was not modeled; moreover, patients whose severe active TAO has not stabilized despite immunosuppression do not proceed to ¹³¹I at our center and are therefore absent from this cohort. The cohort thus still under-represents the most active TAO, and these findings may not generalize to patients receiving ongoing pre-¹³¹I immunosuppression. Fifth, screening, LASSO selection, and coefficient estimation were all performed in the same modest sample, so the estimates lack external validation. The nominal adjusted association of thyroid nodule presence with remission (Section 3.2) emerged only after model fitting and requires confirmation in independent data. Sixth, remission as defined here reflects the four response categories of the 2021 Chinese nuclear medicine guideline (15), which are conceptually consistent with ATA guidance (14). Follow-up duration varied between patients; although every classification used an assessment at 12 months or later, delayed remission or later-onset hypothyroidism may have been captured unevenly, and a standardized fixed-window endpoint should be preferred in prospective work. Finally, 15 participants were younger than 18 years, in whom CAS and sleep patterns may differ, although excluding them did not materially change the estimates (Section 3.2). Accordingly, these associations should not be interpreted causally, and clinical application requires prospective external validation.

### 4.8 Future directions

Future prospective studies should define the outcome and assessment schedule in advance and record each follow-up component. They should retain pretreatment timing for sleep and CAS, add objective sleep measures, and incorporate administered ¹³¹I activity and related dosimetry covariates into multivariable models, together with any concomitant treatment. Clinically justified confounders should be specified beforehand. Any candidate threshold needs external validation before interventions are tested.

## 5 Conclusions

In this single-center retrospective cohort, longer pretreatment nocturnal sleep and lower CAS were associated with thyroid functional remission after ¹³¹I therapy, with consistent directions across sensitivity analyses applying Firth bias reduction, adjusting for age, sex, and smoking, adding thyroid nodule presence, and excluding participants younger than 18 years. Given the retrospective design, unmeasured confounding (including administered activity, thyroid volume, and radioiodine uptake), and post-selection estimation, these associations do not establish causality and require prospective external validation. The absence of remission below 6h of sleep and the fitted inflection points are exploratory *post hoc* observations rather than validated clinical thresholds, and the data cannot show that extending sleep improves ¹³¹I response. Mutually adjusted splines supported a non-linear pattern for CAS but not for sleep. The study cannot inform the ocular prognosis of TAO after ¹³¹I therapy.

## Data Availability

The data generated and analyzed during the current study are not publicly available because they contain sensitive patient information that could compromise participant privacy. Anonymized data may be available from the corresponding author upon reasonable request and subject to ethical approval. The analysis code (Python and R scripts) is available from the corresponding author on reasonable request.
